# Reprogramming the tumor microenvironment with biotechnology

**DOI:** 10.1186/s40824-023-00343-4

**Published:** 2023-01-31

**Authors:** Minjeong Kim, Na Kyeong Lee, Chi-Pin James Wang, Jaesung Lim, Min Ji Byun, Tae-Hyung Kim, Wooram Park, Dae-Hwan Park, Se-Na Kim, Chun Gwon Park

**Affiliations:** 1 Department of Biomedical Engineering, SKKU Institute for Convergence, Sungkyunkwan University (SKKU), Suwon, Gyeonggi 16419 Republic of Korea; 2 Department of Intelligent Precision Healthcare Convergence, SKKU Institute for Convergence, Sungkyunkwan University (SKKU), Suwon, Gyeonggi 16419 Republic of Korea; 3 School of Integrative Engineering, Chung-Ang University, 84 Heukseok-Ro, Dongjak-Gu, Seoul, 06974 Republic of Korea; 4 Department of Integrative Biotechnology, College of Biotechnology and Bioengineering, Sungkyunkwan University (SKKU), Suwon, Gyeonggi 16419 Republic of Korea; 5 Department of Engineering Chemistry, Chungbuk National University, Cheongju, Chungbuk 28644 Republic of Korea; 6 Department of Industrial Cosmetic Science, College of Bio-Health University System, Chungbuk National University, Cheongju, Chungbuk 28644 Republic of Korea; 7 Department of Synchrotron Radiation Science and Technology, College of Bio-Health University System, Chungbuk National University, Cheongju, Chungbuk 28644 Republic of Korea; 8 LANG SCIENCE Inc., Chungbuk National University, Cheongju, Chungbuk 28644 Republic of Korea; 9 Research and Development Center, MediArk Inc., Cheongju, Chungbuk 28644 Republic of Korea; 10 Biomedical Institute for Convergence at SKKU (BICS), Sungkyunkwan University, Suwon, Gyeonggi 16419 Republic of Korea; 11 Center for Neuroscience Imaging Research, Institute for Basic Science (IBS), Suwon, Gyeonggi 16419 Republic of Korea

**Keywords:** Tumor microenvironment, Reprogramming, Combination treatment, Nanoparticle, Biomaterials

## Abstract

The tumor microenvironment (TME) is a unique environment that is developed by the tumor and controlled by tumor-induced interactions with host cells during tumor progression. The TME includes immune cells, which can be classified into two types: tumor- antagonizing and tumor-promoting immune cells. Increasing the tumor treatment responses is associated with the tumor immune microenvironment. Targeting the TME has become a popular topic in research, which includes polarizing macrophage phenotype 2 into macrophage phenotype 1 using Toll-like receptor agonists with cytokines, anti-CD47, and anti-SIPRα. Moreover, inhibiting regulatory T cells through blockades and depletion restricts immunosuppressive cells in the TME. Reprogramming T cell infiltration and T cell exhaustion improves tumor infiltrating lymphocytes, such as CD8^+^ or CD4^+^ T cells. Targeting metabolic pathways, including glucose, lipid, and amino acid metabolisms, can suppress tumor growth by restricting the absorption of nutrients and adenosine triphosphate energy into tumor cells. In conclusion, these TME reprogramming strategies exhibit more effective responses using combination treatments, biomaterials, and nanoparticles. This review highlights how biomaterials and immunotherapy can reprogram TME and improve the immune activity.

## Introduction

Cancer is considered one of the most critical and fatal diseases [1, 2]. Many therapeutic strategies have been developed and tested based on the different types of tumor [3]. In the early stage of development of the therapeutic strategies, tumor removal surgery was the only method available to physically treat cancer patients. Chemotherapy has been investigated since the early days. It is a tumor treatment method that uses drugs to kill tumor cells. Various types of drugs have been discovered and used for tumor treatments. In addition to chemotherapy, one of the most widely used therapeutic methods is radiotherapy [4], which can be used to measure the size of each tumor. Recently, immunotherapy has also become a popular tumor treatment method [5]. Immunotherapy for cancer involves using and controlling the immune system of cancer patients to kill cancer cells. Immunotherapy has the potential to induce durable responses; however, the rates of such responses in patients receiving immunotherapy have been low [6]. Thus, combinations of epigenetics and immunotherapy or biomaterials and immunotherapy has been proposed currently [7].

One of immunotherapy is the reprogramming of the tumor immune microenvironment with various biomaterials. Various categories of biomaterials are used in cancer treatment, currently. Especially, Yang et al. classified novel biomaterials in three categories, which are lipid-based, polymer-based and inorganic biomaterials [8]. Biomaterials are applied in cancer treatment due to therapeutic benefits such as increased biocompatibility, biodegradability, controlled drug release and drug encapsulation efficiency.

Lipid nanoparticles are classified as liposomes, solid lipid nanoparticles, and nanostructured lipid carriers [9]. Liposomes have been taken account of promising materials for drug delivery system [10]. Liposomes are made of phospholipid bilayer with aqueous core. Solid lipid nanoparticles are crystallized particles which are made of fatty acid chains and drug or other molecules [11]. Lipid nanoparticles which act as vehicles contains not only drug, but also protein and nucleic acid [10, 12].

Polymer-based nanoparticles consist of micelles, polymeric nanoparticles, or hydrogels [8]. Micelles have been considered as one of the interesting drug delivery systems for increasing hydrophilic solubility of hydrophobic drugs and enhancing the stability of drug-carriers [13]. Micelles are produced in diameter range between 10 and 100 nm. Polymers such as poly (lactic-co-glycolic acid), poly (ε-caprolactone) and poly (lactic acid) are frequently used to form the hydrophobic core. Furthermore, those polymers are used to generate nanoparticles [14]. Polymeric nanoparticles have wider size range between 1 to 1000 nm. Moreover, the polymer-based biomaterials are usually combined with polyethylene glycol (PEG), which rises blood circulation time and coated biomaterials to improve the tumour targeting [13]. Hydrogels are used in drug delivery for cancer and infection treatments [15]. Drug delivery with hydrogels can control the therapeutic agents release spatially and temporally, and not only small sized molecule drugs, but also macromolecular drugs can be loaded in hydrogels [16]. Furthermore, hydrogels are directly injectable in targeted tumour microenvironment, and improve the immune activity or responses in immunotherapy.

Nanoparticles which are made of metal and non-metal, such as silica, iron oxide or gold are classified as inorganic biomaterials [9]. Inorganic nanoparticles are hydrophilic and highly stable, and reduce frequency of drug doses to lower toxicity of drugs by controlling degradation [17, 18]. Gold nanoparticles and silver nanoparticles are generally used as metallic nanoparticles in cancer treatment. Non-metallic nanoparticles include silica and iron oxide nanoparticle, which are mentioned in this review.

The tumor microenvironment (TME) is a unique environment formed by the tumor and controlled by tumor-induced interactions with host cells during tumor progression [19]. The TME consists of tumor, immune, and stromal cells [20]. Immune cells are essential components of the TME. Interactions between tumor and immune cells have major roles to play in the TME and tumorigenesis [20, 21]. Immune cells can be classified into adaptive and innate immune cells. Adaptive immune cells include T, B, and natural killer (NK) cells [22].

**Scheme 1:**
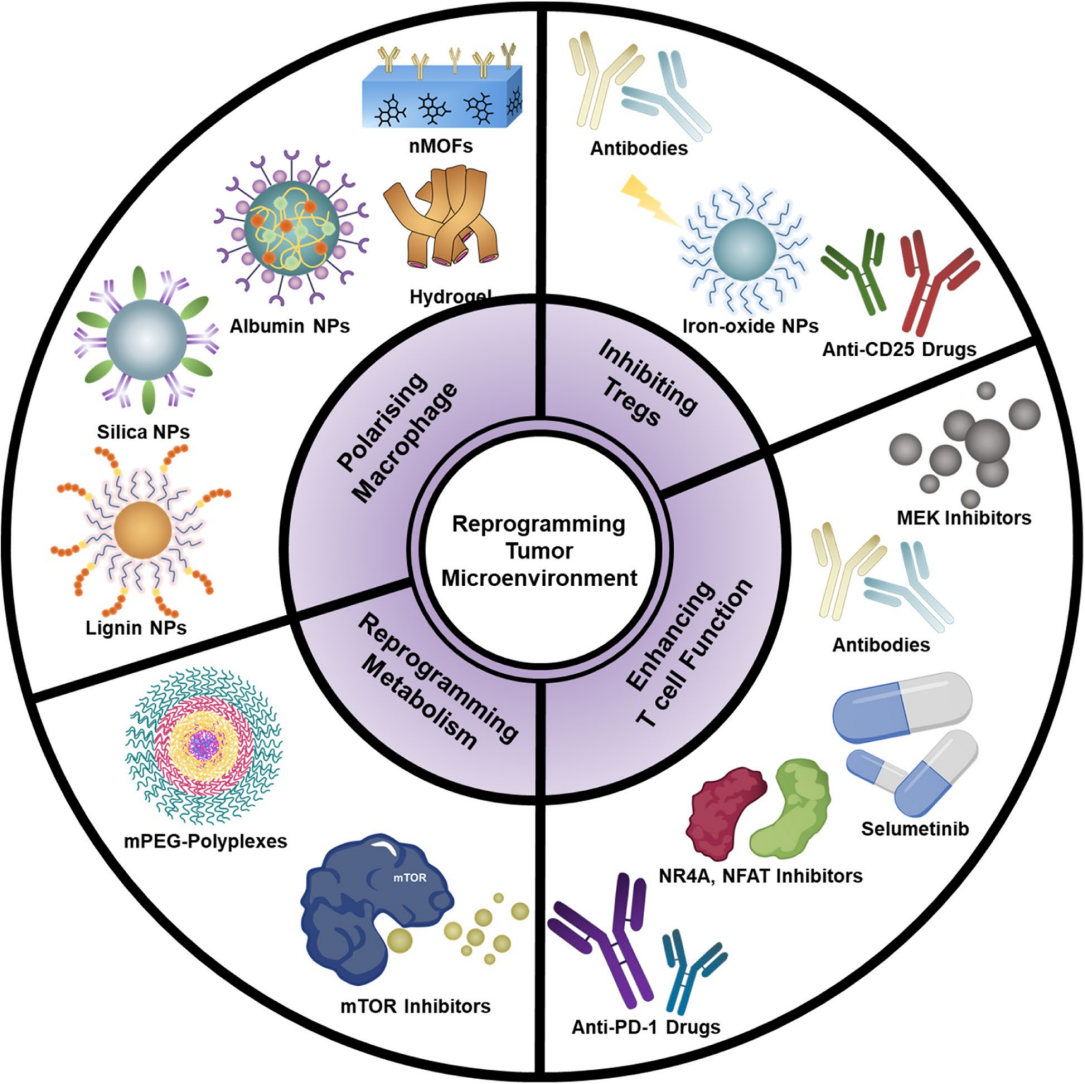
Schematics of various methods of reprogramming the tumor microenvironment

However, not all immune cells are tumor-antagonizing. A few immune cells promote the tumor, thus inhibiting immune activity. To overcome these limitations, researchers have considered the reprogramming of the TME for effective immunotherapy using diverse biomaterials (Scheme 1).

## Tumor microenvironment

The TME is a unique environment that is formed by the tumor and controlled by tumor-induced interactions with host cells during tumor progression [19]. Tumor cells stimulate major molecular, cellular and physical alterations in their host tissues [20]. The TME consists of tumor, immune, and stromal cells [23]. Stromal cells have vascular endothelial cells, pericytes, adipocytes, and fibroblasts [24]. Moreover, they secrete growth factors and cytokines, which affect angiogenesis, proliferation, invasion, or metastasis [21]. Therefore, stromal cells and tumor cells promote cancer progression, proliferation, and growth.

Immune cells are essential components of the TME. Interactions between tumor and immune cells play a major role in the TME and tumorigenesis [20, 21]. Immune cells can either repress tumor progression or promote tumor growth. Generally, immune cells can be classified into adaptive and innate immune cells. Adaptive immune cells include T, B, and NK cells [22]. They respond slowly with pathways that recognize cancer indirectly. In contrast, innate immune cells include macrophages, neutrophils, and dendritic cells (DCs). They respond rapidly with pathways that recognize cancer directly. However, immune cells associated with the tumor can be classified into two types: tumor- antagonizing and tumor-promoting immune cells [23, 25]. The main effector T cells, NK cells, DCs, and macrophages comprise the tumor-antagonizing immune cells, whereas the regulatory T cells (Tregs) comprise the tumor-promoting immune cells [26]. These different types of immune cells serve their functions at each stage of tumor formation. In the early stage, which is the tumor initiation stage, effector immune cells are expressed to eliminate the initial tumor cells. Primarily, effector T cells, macrophage phenotype 1 (M1), and NK cells respond at the initial stage. Effector T cells contain cluster of differentiation (CD) 8^+^ T, cytotoxic T, and effector CD4^+^ T cells. As the tumor cells grow, in a later stage, known as metastatic dissemination, the immunosuppressive cells in the TME, such as Tregs, macrophage phenotype 2 (M2), and immature DCs, are primarily expressed.

### Major tumor-antagonizing immune cells

Tumor-antagonizing immune cells are likely to kill cancer cells in various stages of tumorigenesis within TME [23]. Whereas, tumor-promoting immune cells tend to inhibit response of tumor-antagonizing immune cells, and support tumorigenesis.

CD8^+^ T and NK cells are the most important lymphocytes for recognizing and eliminating tumor cells. CD8^+^ T cells, which are activated and contributed by helper CD4^+^ T cells (Th1 CD4^+^ cells), which produce interleukin (IL)-2 and interferon (IFN)-ɣ [27]. IL-2 strengthens CD8^+^ T cell proliferation, and IFN-ɣ induces cytotoxicity in tumor cells and stimulates M1 to exert anti-tumoral effects. Thus, the increased levels of Th1 CD4^+^ cells in the TME are associated with cancer. CD8^+^ T cells kill tumor cells through granular exocytosis and apoptosis. In addition, they cause cytotoxicity in tumor cells by producing IFN-ɣ and tumor necrosis factor (TNF)-α. Each T cell improves the activity of its receptor that recognizes a definite antigen. In other words, CD8^+^ T cells detect abnormal tumor antigens in tumor and cancer cells to destroy them. After their activation, the programmed death-1 receptor (PD-1) may be expressed for a short duration by the activated T cells.

NK cells are essential innate tumor-antagonizing lymphocytes that control the immunosuppression by a tumor, and they play a similar role as CD8^+^ T cells [27]. NK cells produce pro-inflammatory cytokines and chemokines to improve their anti-tumor activity. Moreover, they express major histocompatibility complexes (MHC)-1-specific inhibitory receptors to eliminate MHC-1-deficient tumor cells [28]. However, certain adverse effects of NK cells acting as anti-tumor immune cells by regulating DCs and T cells have been reported recently. NK cells can be classified into two types according to the functions they perform. These are killing tumor cells and secreting inflammatory cytokines, such as IFN-ɣ, TNF, and GM-CSF, to promote anti-tumor activity [28]. In other words, most NK cells kill tumor cells; however, only a few NK cells kill the TME.

DCs are mainly considered to be the antigen-presenting cells (APCs) in the TME. They present antigens and provide the necessary costimulatory signals and cytokines for T cell activation. DCs acts as a connection between innate and adaptive immunity. During tumor development, DCs can cause priming of the naive and memory T cells. Furthermore, they can cause priming and initiating of the effector T cell response or antigen tolerance, dependent on the costimulatory signals and the inflammatory conditions [29]. Tumor-infiltrating DCs play a key role in describing the function of T cells during tumor progression.

A macrophage is a primary immune component of the innate immunity and is derived from circulating monocytes within the TME. The macrophage is divided into two polarizations: M1 and M2 [26]. M1 is an anti-tumor and pro-inflammatory macrophage, and it has a critical role to play against pathogens driven by cytokines, such as IFN-ɣ and TNF-α [30].

### Major tumor-promoting immune cells

In contrast, M2 is an anti-inflammatory and pro-tumorigenic type of macrophage and causes tumor promotion and causes immunosuppression in the TME [30]. Moreover, M2 primarily inhibits the functions of T cells, and reduces cancer-associated inflammation. Thus, the activity of M2 produces anti-inflammatory cytokines, causes immunosuppression in the TME, and contributes to angiogenesis, tumor progression, and metastasis.

One of main type of tumor-promoting immune cells is Tregs [23]. Tregs act as a mediator between the control of autoimmunity and suppression of inflammatory responses. As the number of Tregs increase in the TME, they appear to be associated with an improved outcome. Thus, Tregs have been targeted as biomarkers in the TME.

However, an excessive increase in the number of Tregs can result in the change of immunosuppression to normal immunity in the TME. Therefore, Tregs promote tumor development or progression and exhibit anti-tumor immune responses by secreting IL-10 and TGF-β [26, 31]. Tregs maintain the homeostasis of cytotoxic lymphocytes by mediating the expansion and activation of T cells. TGF-β supports the immunosuppressive responses of Tregs. TGF-β signaling typically maintains cell homeostasis by controlling cell proliferation and apoptosis. However, fibrosis or cancer can occur when the TGF-β signaling is disturbed. At the tumor initiation stage, TGF-β suppresses cancer. In contrast, in the final stage of tumor progression, TGF-β promotes cancer. IL-10 is considered to be a mediator in the suppression of Tregs. Moreover, it inhibits the maturation of DCs to accelerate tumor growth during tumorigenesis. The TME has a vital role in tumor epigenetics, differentiation, immune evasion, and dissemination [28]. The TME is characterized by many mechanisms supporting angiogenesis and immunosuppression [31].

**Fig. 1:**
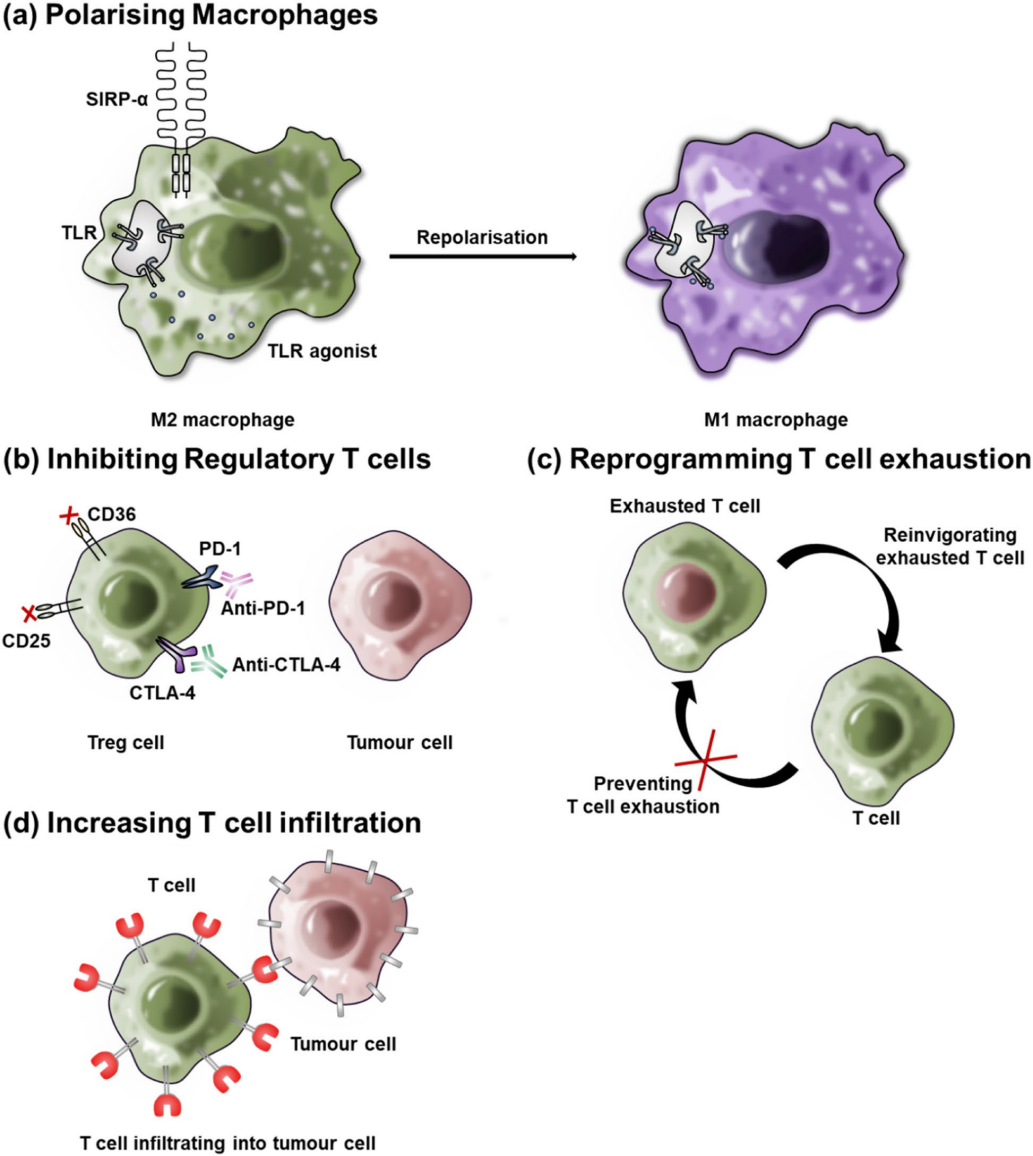
Schematic of tumor microenvironment reprogramming with biomaterials. **a** Repolarizing the M2 into the M1, **b** inhibiting regulatory T cells (Tregs), **c** reprogramming T cell exhaustion, **d** enhancing T cell infiltration by reprogramming

Several TME reprogramming methods exist, such as macrophage repolarization (Fig. 1a), Tregs inhibition (Fig. 1b), reprogramming T cell exhaustion (Fig. 1c), T cell infiltration (Fig. 1d), and targeting metabolic pathways. Our review presents an introduction to the reprogramming of the TME with combination treatment, biomaterials, and nanoparticles.

## Reprogramming the tumor microenvironment

Most cancer treatment methods directly strengthen the function of immune cells; in other words, immunotherapy improves immune responses. A method of correcting abnormal TME (TME reprogramming) that inhibits immunosuppression from occurring, is considered an attractive method to improve the outcome of immunotherapy. Here, we have reviewed the following TME reprogramming methods: Tumor-associated macrophages, Tregs, T cell exhaustion, T cell infiltration, and metabolic pathways.

### Macrophage polarization

M1 and M2 are crucial for maintaining tissue homeostasis and recovering tissues [32]. Generally, M1 infiltrates inflammatory tissues for treatment and M2 appears after M1 to advance anti-inflammatory interactions, such as tissue repair. However, in the TME, M1 is an anti-tumor phenotype and M2 is a pro-tumor phenotype [33]. The ratio of M1 and M2 indicates the condition of the TME, as M1 is associated with a positive outcome and M2 is associated with an unfavorable survival of the tumor cells [34]. The polarization of tumor-associated macrophages (TAMs) is controlled by tumor cells in TME, and an increase in the M1/M2 ratio is associated with an improved prognosis [29, 33]. Therefore, the polarization of M2 into M1 has been investigated.

#### Toll-like receptor (TLR) agonists with cytokines

Macrophage polarization is related to the stimulations expressed by TLR agonists or cytokines [35]. TLR agonists play a vital role in the innate immune system and can induce an immune response.

TLR 7/8 stimulates innate immune cells, resulting in the activation of humoral and cellular immunity [29]. It activates the immune cells and promotes inflammation [29, 36–38]. Therefore, it engenders a series of anti-tumor activities. Figueredo et al. researched the reprogramming of M2 macrophages into M1 using a nanomedicine [38]. The nanomedicine was created from R848, which is a TLR7/8 agonist, and lignin nanoparticles (LNPs). LNPs redesign the biodistribution of R848 and target CD206-positive M2-like macrophages. R848-loaded LNPs (R848@LNPs) are delivered to reprogram M2 into M1-like macrophages using mUNO, which is CD206-targeting peptide, increasing the effects of empty LNPs or R848@LNPs with M2 in TME.

**Fig. 2:**
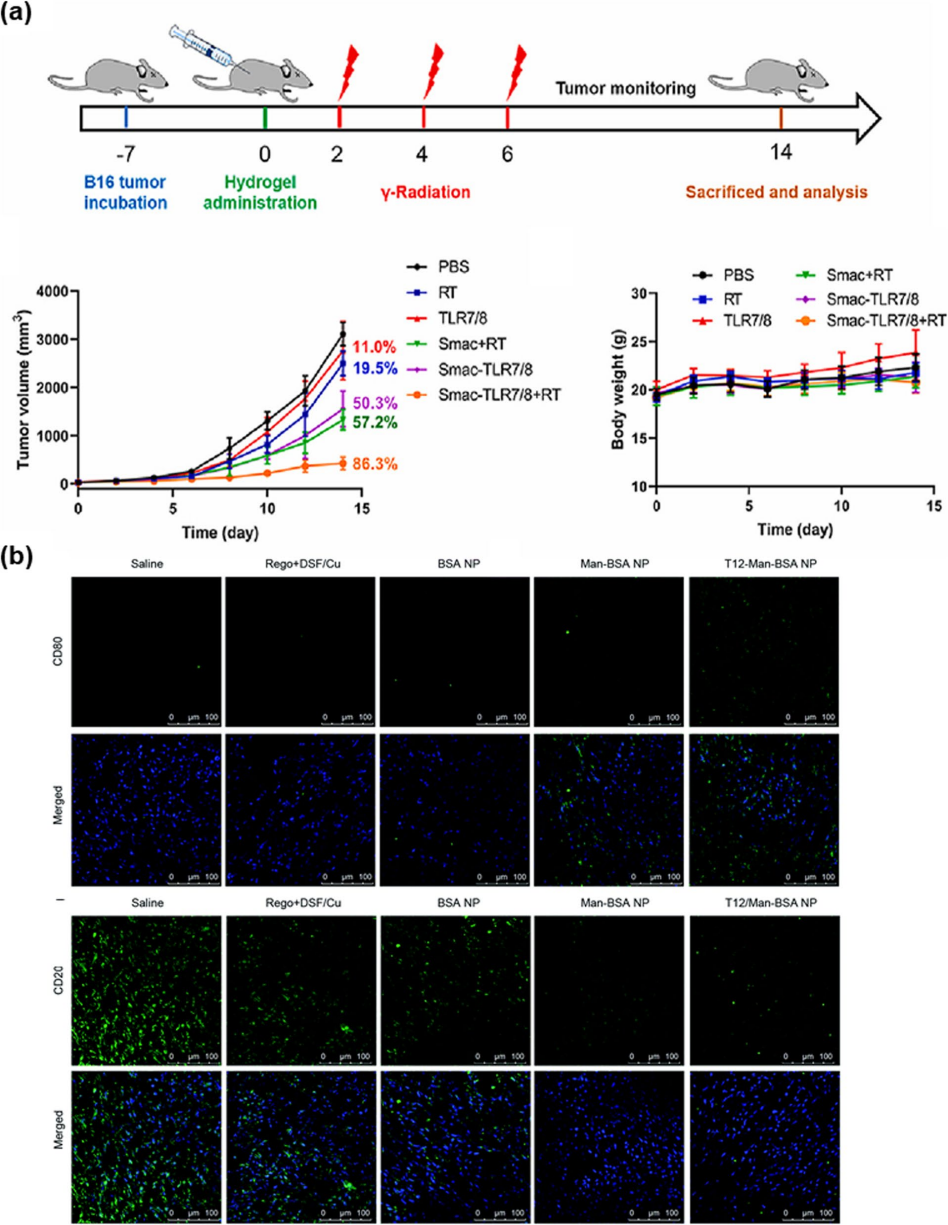
TLR7/8 agonists can decrease tumor growth. **a** In vivo tumor treatment of Smac-TLR7/8 hydrogels during radiotherapy. Tumor volume curves in general and body weight of different treatment groups. (Reproduced with permission from [29] Copyright 2022, Bioactive Materials). **b** Expression of CD80 (M1) (upper) and CD206 (M2) (below) in U87 orthotopic glioma of Balbc/nude mice after treatment. Albumin-binding nanoparticles reprogram M2 into M1. (Reproduced with permission from [39] Copyright 2018, Chemical Science)

R848 loaded on β-cyclodextrin (CD), which has hydrophilic outers shell and provides the hydrophobic cavity, nanoparticles (CDNP-R848) was investigated for effective drug delivery to TAMs. The delivery of CDNP-R848 to TAMs exhibited an increase in the production of IL-12 (produced by the innate immune system) and promoted the production of cytokines associated with the anti-tumor activity (Fig. 5a) [36]. In other words, M2, which is known as pro-tumor macrophage was reprogrammed as anti-tumor macrophages, M1. Moreover, Rodell et al. observed that the immune response increased when using CDNP-R848 with anti-PD-1 [37]. The CDNP could not affect the tumor growth by itself. However, the combination of anti-PD-1 and CDNP-R848 was mutually beneficial as it decreased tumor area. Moreover, Zhang et al. observed that using radiosensitive peptide hydrogel conjugated with TLR7/8 (Smac-TLR7/8 hydrogel) can regulate M2 polarization into M1 (Fig. 2a) [29]. After repolarization, the anti-tumor immune response was activated, and the growth of the tumor decreased. The bioactivity of TLR7/8 improves with an increase its stability and availability. As a result, the Smac-TLR7/8 hydrogel improved the anti-tumor activities of the macrophages, directed the phagocytosis of tumor cells, and increased the secretion of TNF. In other words, the Smac-TLR7/8 hydrogels could repolarize M2 into M1. Furthermore, the Smac-TLR7/8 hydrogel and anti-PD-1 with radiation could boost the immune responses. Anti-PD-1 increased the infiltration of tumor lymphocytes and decreased the activity of Tregs. A combination treatment of immunotherapy and chemotherapy using nanoparticles has been used. Zhao et al. developed the albumin nanoparticle with dual binding ligands, a transferrin receptor, and SPARC, an albumin-binding receptor on tumor cells. This albumin-binding nanoparticle targets mannose receptors on M2 and pro-tumor M2 in patients with glioma and inhibits the glioma cell growth and proliferation by reprogramming pro-tumor M2 into anti-tumor M1-like macrophages (Fig. 2b) [39].

**Fig. 3:**
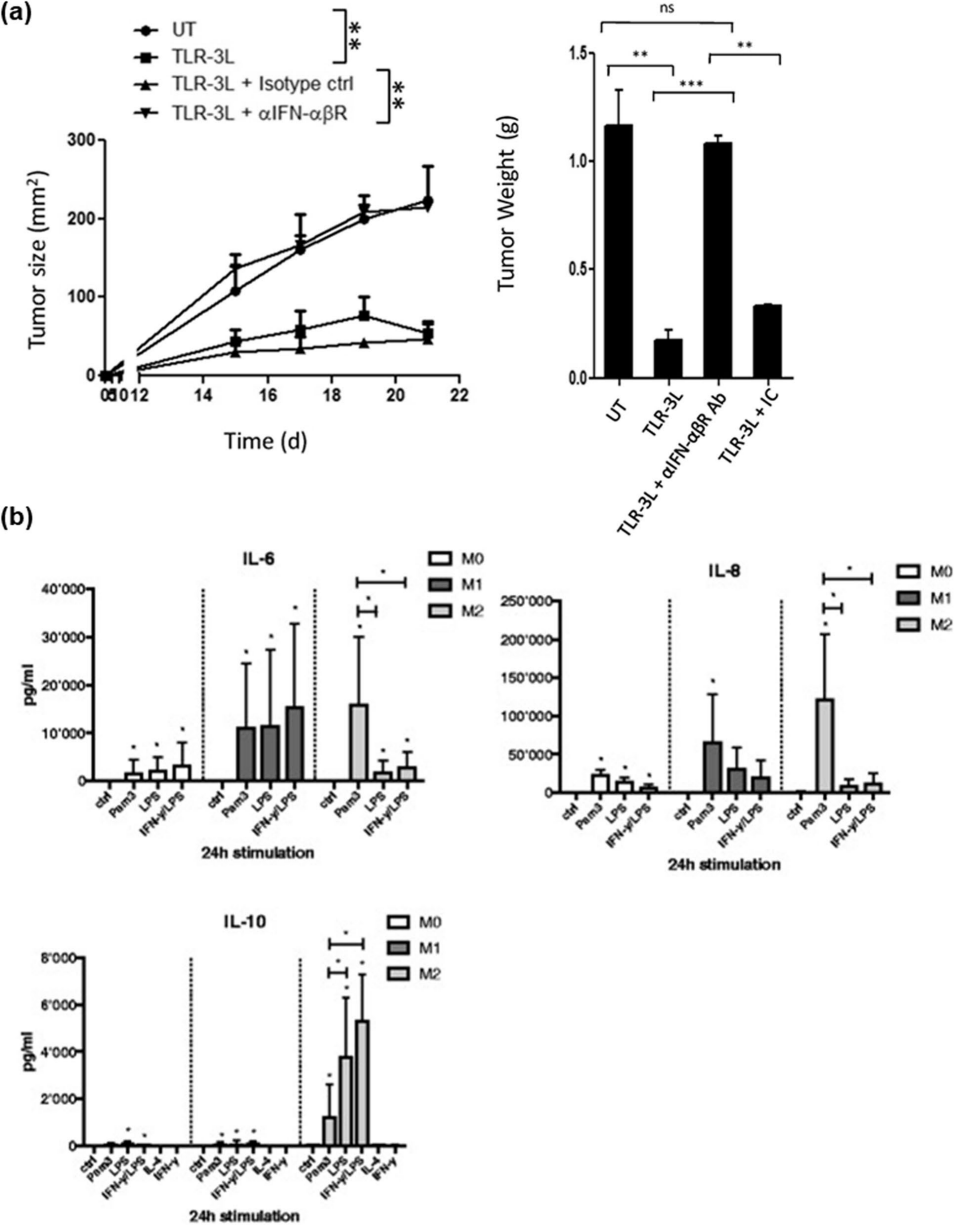
TLR2 and TLR3 can convert M2 into M1. **a** TLR-3 triggering reverts human M2 to M1. The size of the tumor is indicated in squared millimeters at different time points. (Reproduced with permission from [40] Copyright 2018, Frontiers in Immunology). **b** Cytokine profile of M0-, M1-, and M2-polarized macrophages following TLR ligand exposure and activation. (Reproduced with permission from [43] Copyright 2017, Arthritis Research & Therapy)

Recently, TLR3 has been considered to play a major role in cancer immunotherapy. TLR3 stimulates M2 to change into M1 at certain levels of mRNA and protein. Vidyarthi et al. discovered that TLR3 triggering has no side effects, such as toxicity, and it induces IFN-α [40]. Moreover, they observed that as TLR3 triggers further, M2a and M2c macrophages are reverted into M1. TLR3 signaling inhibits the polarization of the M2a and M2c subtypes with the up-regulation of CD86, an established marker for M1, and down-regulation of CD206 and TIM-3, a marker of the type M2 and the role in the negative regulation of T cell responses, respectively. Research shows that TLR3 changes M2 into M1, and represses the tumor growth (Fig. 3a). Zhao et al. investigated nanoparticles that can repolarize M2 into M1 to treat melanoma and metastasis [41]. The nanoparticles are called FP-NPs {nanoparticles composed of amino-modified ferumoxytol-NH_2_ surface functionalized with Poly [I:C]}. Poly (I:C) (PIC) frequently interacts with TLR3 relevant to an innate immune response [42]. FP-NPs can delay the B16F10 cell growth by repolarizing M2 into M1 via NF-κB signaling [41].

An increase in TLR2 reduces the activity of M2 [43]. Quero et al. observed that TLR2 stimulates chimeric M2, which has been determined by surface markers, to perform an M1-like function, which has been determined by genetic markers and cytokine secretion (Fig. 3b). As evidence of this research, loading more TLR2 demonstrated that the ratio of IL-10 to IL-6 or IL-8, a pointer of an anti-inflammatory cytokine, reduced [44]. Furthermore, TLR2 agonists can be designed to produce anti-tumor potential macrophages, which are M1-like macrophages [45]. Jiang et al. investigated nanoparticles [46]. The nanoparticles were based on chitosan and were prepared to identify their effects on macrophage polarization. After the injection of chitosan nanoparticles (CNs) into the mouse acute lung injury model, the levels of TNF-α and IL-10 increased, and STAT-6 pathways were induced. STAT-6, which is activated by IL-4, can suppress the STAT-1 function. Furthermore, CNs control the homeostasis of the M1/M2 ratio. TLR4 and TLR2 are related to CNs; therefore, the TLR4/TLR2 significantly increased after the injection of CNs. Therefore, CNs can affect macrophage repolarization (Fig. 5b).

#### Anti-CD47 and Anti-SIRPα

CD47 is a membrane glycoprotein signal, which means “do not eat me” on tumor cells [47]. Normal tissue cells exhibit low expression of CD47 [48]. CD47 controls physiological functions, such as cell growth, cell migration, cytokine production, and T cell activation [49]. Signal regulatory protein alpha (SIRPα) is an immune receptor in macrophage cells, which interacts with CD47 in the TME. The interaction between CD47 and SIRPα eludes recognition and prevents innate immune response [48]. Therefore, the blockade of the interaction between CD47 and SIRPα is considered a potential strategy to reactivate the phagocytic immune activity of macrophages [47].

**Fig. 4:**
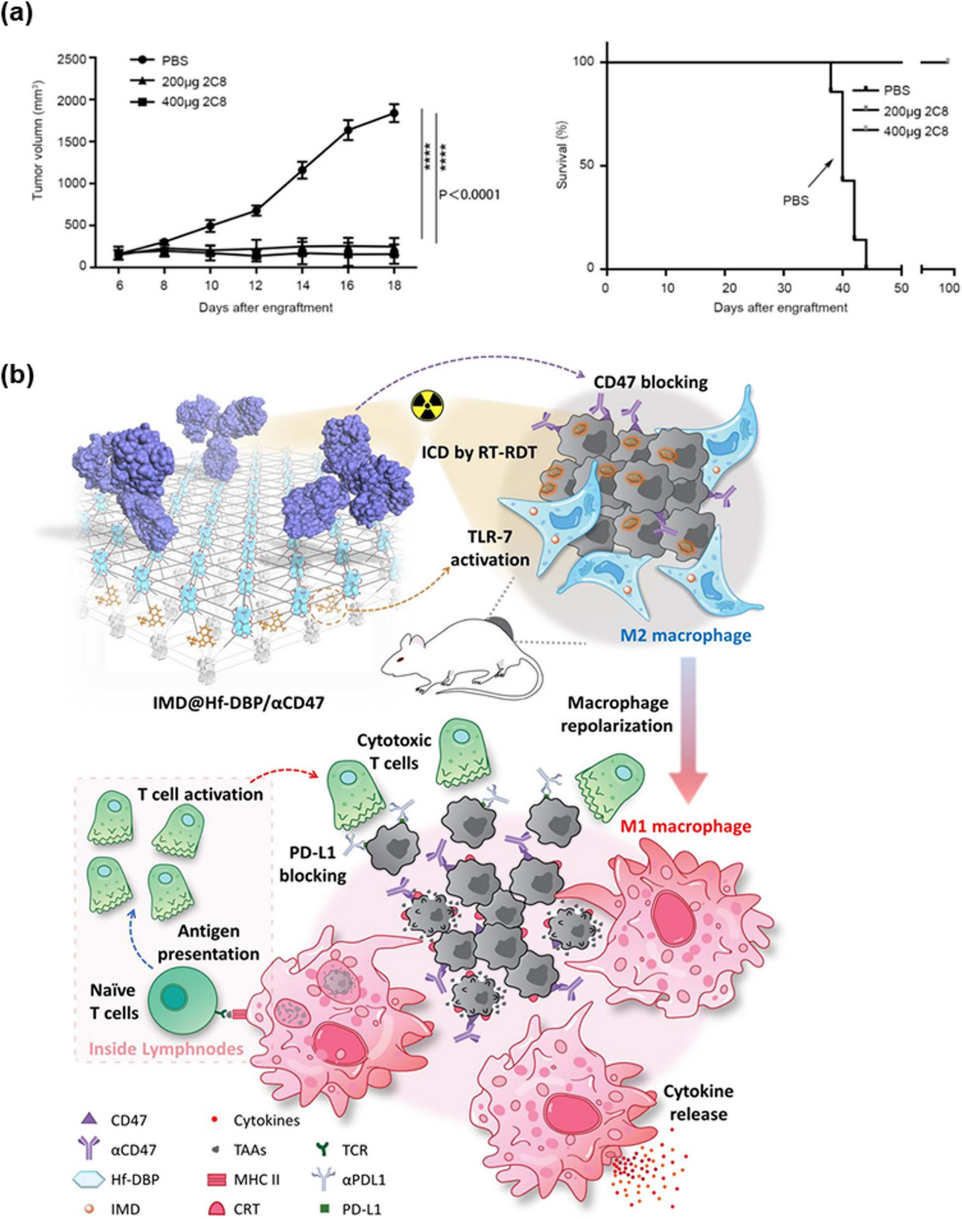
Targeting CD47 and SIRPα with nanoparticles. **a** 2C8 inhibits tumor growth in xenotransplantation models. Mice were treated with two different doses of 2C8 or Phosphate-buffered saline. The tumor volume of tumors per group is depicted over time. (Reproduced with permission from [53] Copyright 2020, Frontiers in Oncology). **b** Schematic showing repolarization of M2 to M1 and promoting phagocytosis by blocking the signal in tumor cells by IMD@Hf-DBP/αCD47 and X-ray radiation (Reproduced with permission from [54] Copyright 2020, American Chemical Society)

Targeting CD47-SIRPα, which functions as an immune checkpoint, includes using anti-CD47 or anti-SIRPα antibodies [50, 51]. Clinically, Zhang et al. observed that when anti-CD47 inhibited the interaction of CD14-SIRPα, M1-like macrophages in most tumor cells increased and became higher than M2 macrophages [52]. Moreover, CD47-SIRPα blockade can result in increased anti-tumor activities [47] and enhanced macrophage phagocytosis of the tumor cells (Fig. 4a) [53]. Targeting CD47-SIRPα promotes macrophage migration into the TME, and causes TAMs to attack tumor cells by changing TAMs from M2 to M1-like macrophages [50, 53]. Lin et al. studied the CD47 blocking antibody, which is called 2C8, for inhibiting CD47-SIRPα interaction. 2C8 has high specificity and affinity of CD47 protein and suppresses tumor growth. Furthermore, blocking CD47 with 2C8 resulted in an increased number of M1-like macrophages in the TME [53].

**Fig. 5:**
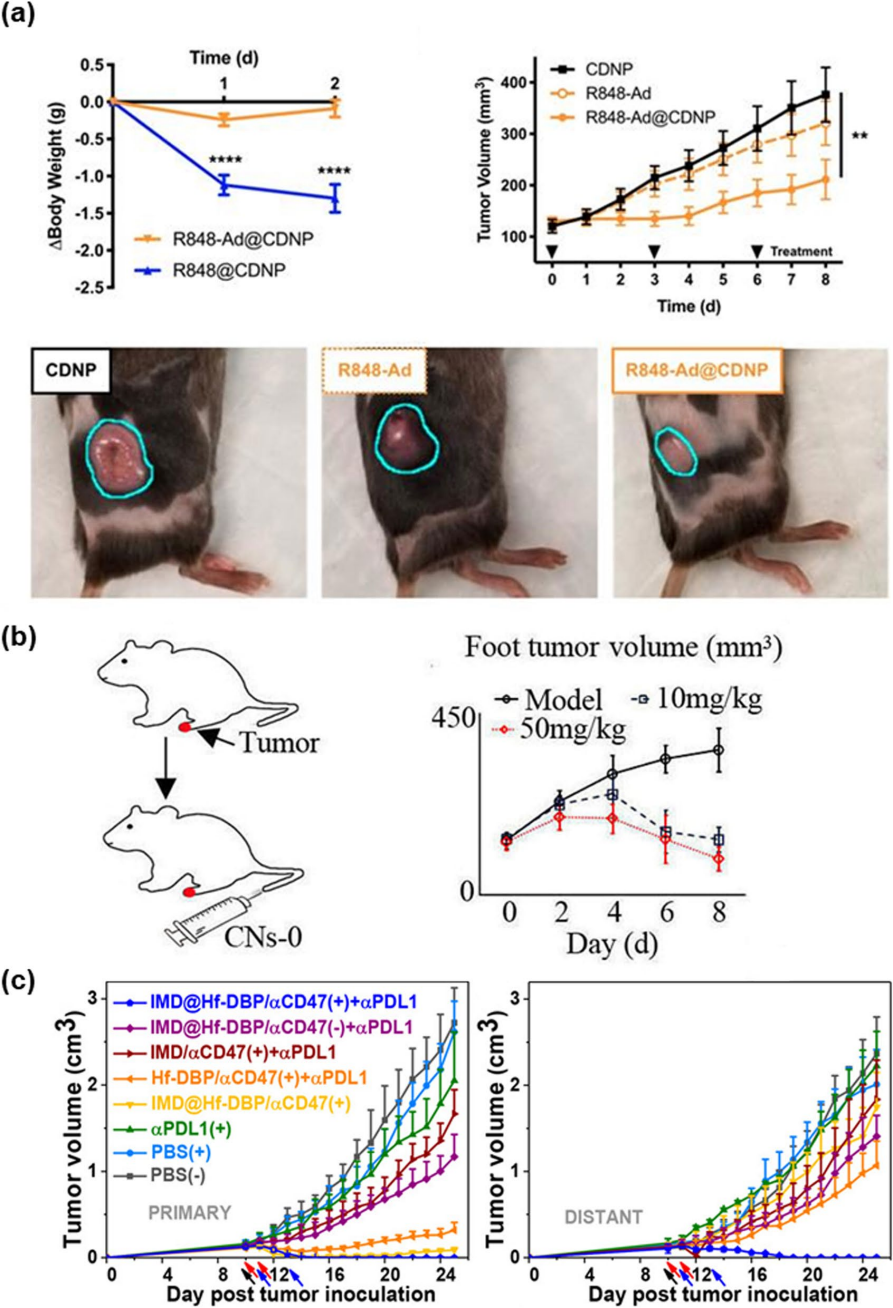
Reprogramming of TAM with biomaterials. **a** A combination of R848, which is a TLR7/8 agonist, and CDNP can decrease the tumor size. (Reproduced with permission from [36] Copyright 2019, Theranostics). **b** Effect of chitosan nanoparticles on reprogramming of TAMs and tumor metastasis in animals, the mouse acute lung injury model, was established (Reproduced with permission from [46] Copyright 2022, Elsevier). **c** Growth curves of primary tumors and distant tumors of bilateral CT26 tumor-bearing mice. Black, red, and blue arrows refer to intratumoral injection, X-ray irradiation, and intraperitoneal injection, respectively. (Reproduced with permission from [54] Copyright 2020, American Chemical Society)

In summary, some nanoparticles block the CD47-SIRPα interaction. The nanoparticles can engineer TAMs by inhibiting recruitment, depleting TAMs, and reprogramming TAMs [55]. For example, Zhang et al. researched a pro-phagocytic nanoparticle, called SNPA _calr&acd47_, which carries the CD47 antibody and the pro-phagocytic molecule calreticulin (CALR) and regulates macrophage phagocytosis [56, 57]. Moreover, Ni et al. discovered a co-delivery system using anti-CD47 antibodies and TLR-7 agonists to reprogram TAMs [54]. The nanoscale metal–organic framework (nMOF) is used with radiotherapy to increase its effectiveness. Ni et al. created the nMOF, called IMD@Hf-DBP/αCD47 (Fig. 4b), a combination of anti-CD47 antibody, nMOF and imiquimod that activates the TLR-7 pathway (Fig. 5c). The therapeutic strategies and delivery methods, which are used for reprogramming TAMs, are organized in Table 1.

**Table 1:** Macrophage polarization with biomaterials

Type	Load	Delivery Materials	Therapy	Injection	Targeted protein	Ref
Toll-like Receptor	R848	Lignin nanoparticles	in vivo	intraperitoneal injection	CD206	[38]
		CDNP-R848 with anti-PD-1	in vivo	intravenous injection	-	[36, 37]
	TLR7/8 agonist	Smac-TLR7/8 hydrogels with anti-PD-1	in vivo	peritumoral injection	CD86 andCD206	[29]
	TLR3	-	in vitro		CD86, CD206, and TIM-3	[40]
		FP-NPs	in vivo	intravenous injection	-	[41]
	ES antigen	-	in vitro		CD11b	[44]
	Pam3, IFN-γ, and LPS	-	in vitro		CD14 and CD163	[43]
	-	Chitosan nanoparticle	in vivo	intravenous injection	-	[46]
CD47	CD47-blocking antibody (2C8)	-	in vivo	intravenous injection	CD47	[53]
	Anti-CD47 antibody	-	in vivo	intraperitoneal injection	CD47	[52]
	CD47-blocking antibody and CALR	Silica nanoparticle	in vivo	intratumoral injection	CD47	[57]
	Anti-CD47 and Imiquimod	IMD@HfDBP/αCD47 (nMOF)	in vivo	intratumoral injection	CD47	[54]
Albumin	TfR, SPARC	Albumin nanoparticle	in vivo	intravenous injection	-	[39]

### Inhibiting regulatory T cells

Tregs, a subset of CD4^+^ T cells, are potential immunosuppressive cells in the TME [58, 59]. They suppress inflammatory activity, result in tumor growth, and boost immune evasion by tumor cells [60]. FOXP3 regulates Tregs functions, and suppresses anti-tumor immunity [61]. CD36 also modulates immunosuppressive functions of intratumoral Tregs [62]. Increased number of Tregs, detected in TME [63] can be used as therapeutic targets for tumor immunotherapy [60]. Thus, some studies have recently developed strategies to treat tumor cells by blocking the activities of Tregs or by depleting Tregs [59].

### Blocking regulatory T cells

**Fig. 6:**
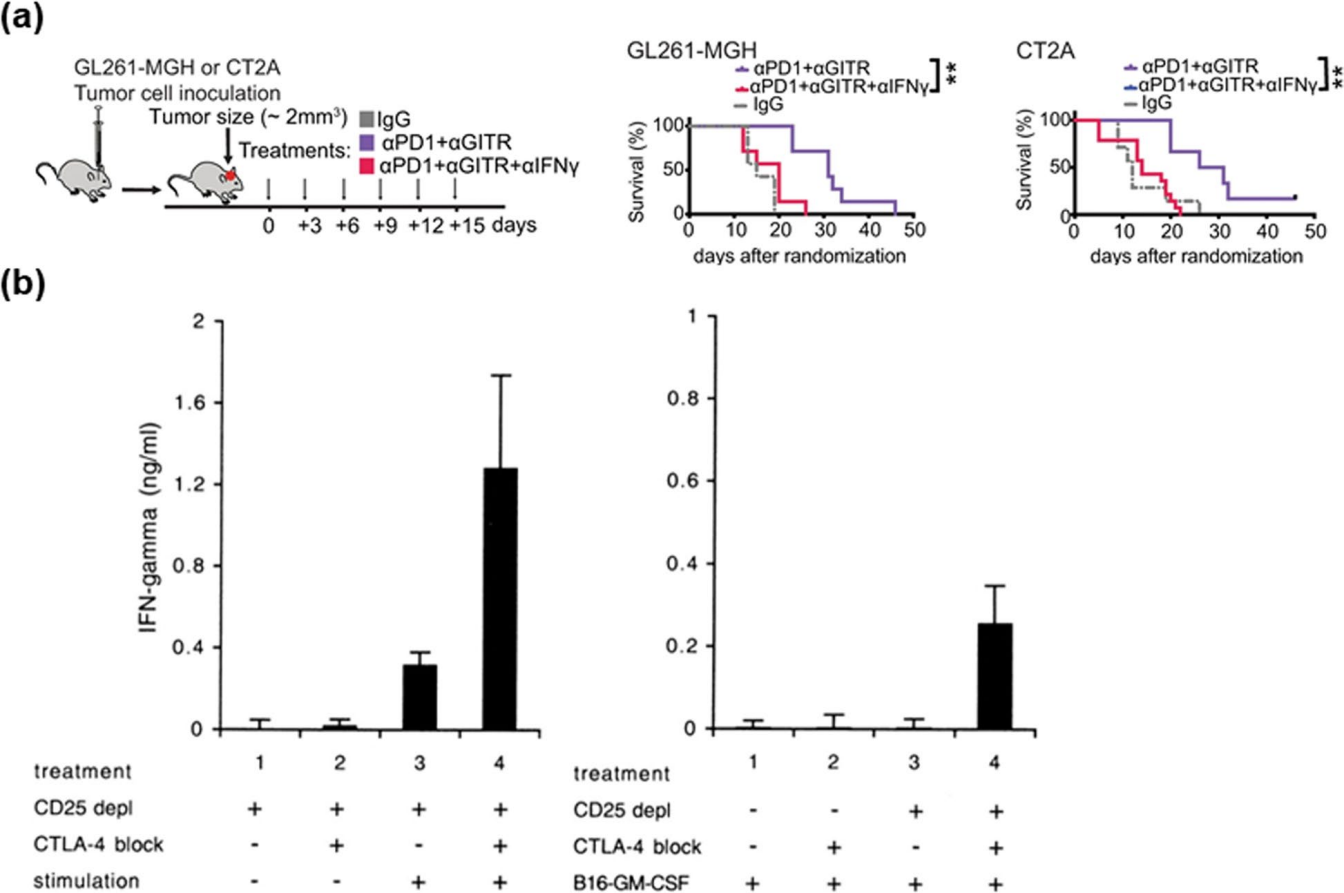
Inhibition and depletion of Tregs in TME can increase the survival rate and decrease tumor growth. **a** Schematic of the experimental setup to evaluate the contribution of IFN-γ to anti-tumor T cell activity in vivo. Mice bearing orthotopic glioblastoma tumors (GL261-MGH or CT2A, size -2 mm^3^) were treated with six doses of (i) αPD1 + αGITR, and (iv) αPD1 + αGITR + αIFN-γ (250 μg/mouse). (Reproduced with permission from [68] Copyright 2021, Nature Communication). **b** CTLA-4 blockade enhances CTL induction in the absence of CD25^+^ Tregs. CD25.^−^ splenocytes were used to analyze the effect of CTLA-4 blockade on the induction of effector CTL in vitro (left) and in vivo (right). (Reproduced with permission from [69] Copyright 2021, Clinical Cancer Research)

Various approaches are used to treat cancer cells through Tregs blockade. Targeting immune checkpoints is one of these therapeutic strategies. Immune checkpoint blockade and inhibition decrease immune suppression induced by tumor cells [64]. Immune checkpoints are selected for blockade because they have negative roles in immune responses and T cell activation. PD-1 and CTLA-4 are the most representative immune checkpoints [59]. High PD-1 expression can be unfavorable in suppressing Tregs activity [65]. Moreover, anti-PD-1 and anti-PD-L1 can promote the anti-tumor activity of CD8^+^ T cells by suppressing Tregs activity. Blocking PD-1 and PD-L1 with antibodies interferes in FOXP3 expression [59, 66]. Thus, controlling PD-1 expression is a potential clinical strategy. CTLA-4 functions as an immune checkpoint and interrupts immune responses and APCs [65, 67]. CTLA-4 also represses the immune responses and promotes the survival of tumor cells [59]. Moreover, it results in Tregs instability in tumors [65]. Thus, anti-CTLA-4 can be used not only for Tregs blockade but also for Tregs depletion [65]. However, another opinion is that the use of anti-CTLA-4 itself may decrease a small amount of Tregs [65, 68]. To supplement this opinion, Amoozgar et al. studied the relationship between anti-CTLA-4 and anti-PD-1 [68]. The administration of anti-CTLA-4 primarily with anti-PD-1 is preferred to reduce Tregs anergy. Moreover, Amoozgar et al. observed that the combination of anti-PD-1 and anti-GITR can convert immunosuppressive Tregs into Th1 effector cells. As a result, the Tregs anergy decreases with reduced production of TGF-β and IL-10, and the secretion of INF-ɣ and TNF-α increases (Fig. 6a).

Chemotherapy and radiotherapy, which are established cancer treatments, can decrease Tregs activity and increase effector T cell activity [70]. However, no significant successful research has been conducted on chemotherapy and radiotherapy for reprogramming of Tregs metabolism. However, Tregs metabolism can be targeted by inhibiting metabolism-related signaling mediators, which include TGF-β and AMPK, in fatty acid (FA) metabolism or amino acid catabolism. Furthermore, negatively, the AKT, PI3K, and mTOR signaling pathways are the main pathways to control metabolism reprogramming and Tregs repression [61]. In other words, the inhibition of metabolic pathways can promote the immunosuppressive Tregs. Previous research has shown that the suppression of PI3K or mTOR complex 1 (mTORC1) result in reduced inhibitory immune checkpoint expression, such as PD-1 and CTLA-4, and has a negative influence on Tregs. Because Tregs express CTLA-4, which has a vital role in FOXP3 expression, targeting CTLA-4 can result in positive Treg reprogramming and inhibit Tregs stability [70].

### Depleting regulatory T cells

Another therapeutic strategy to reprogram Tregs is Tregs depletion [59]. Many researchers have recently addressed this therapy following the Tregs blockade. Tregs depletion has been considered to increase anti-tumor immune responses [71]; however, excessive exhaustion of Tregs can cause over-autoimmunity. Therefore, researchers have suggested practical strategies for reprogramming Tregs [61].

One of those strategies is targeting CD25 for the depletion of CD25^+^ T cells, which has demonstrated increased anti-tumor immune responses [72, 73]. CD25 is the IL-2 receptor α-chain, and Tregs express the IL-2 receptor. As the amount of CD25 decreases, the anti-tumor immune responses of Tregs decreases. Thus, the use of anti-CD25 antibody, called daclizumab, or cyclophosphamide, the chemo-drug, successfully depletes Tregs [67]. Anti-CD25 administration has resulted in improved survival [74, 75].

As discussed in Sect. 3.2.1., anti-CTLA-4 is used to deplete Tregs. Takeuchi et al. observed that the anti-CTLA-4 antibodies exhibit anti-tumor activity depending on the depletion of CTLA-4 Tregs in the TME [67]. Ha et al*.* studied whether the anti-tumor responses of anti-CTLA-4 could deplete Tregs. They attempted to protect CD8^+^ T cells expressing CTLA-4 from killing Tregs using anti-CTLA-4 antibodies (Fig. 6b) [76]. Similarly, recent research showed that modified anti-CTLA-4 decreases antibody-dependent cellular cytotoxicity by modulating the Fc portion or the Fc receptor. Furthermore, its anti-tumor immune responses encourage the depletion of FOXP3^+^CD4^+^ Tregs [73]. Furthermore, anti-CTLA-4 makes FOXP3^+^CD4^+^ Tregs alleviate their immunosuppressive activities and contribute to anti-tumor immune response [66, 72, 73]. Chen et al*.* observed that the combination of iron-oxide nanoparticles-mediated Photothermal therapy and anti-CTLA-4 can deplete Tregs and enhance CD8^+^ T cell activation [77].

Similarly, targeting CD36 can result in Tregs apoptosis [62]. The expression of CD36 causes suppressed immune responses in Tregs and affects the expression of activation markers, such as CD44, CD103, or FOXP3, in intratumoral Tregs. Peroxisome proliferator-activated receptors (PPAR) consist of three receptors: PPAR-α, PPAR-β, and PPAR-γ [78]. Primarily, PPAR-β functions as an energy progression by increasing oxidation and oxidative phosphorylation, it has a role in tumor vascularization in TMEs, which can aid tumor progression [79]. CD36-PPAR-β signaling can support the prolonged survival of Tregs and magnify CD36 metabolism in intratumoral Tregs [62]. The interaction of PPAR-β and CD36 stimulates fatty acid oxidation to maintain the mitochondrial fitness and electron transport chain function. Thus, the depletion of CD36 can result in the apoptosis of Tregs with decreasing tumor growth [80]. Targeting CD36 to block metabolic adaptation can inhibit Tregs immunosuppressive functions with minor loss [62]. Moreover, it can provide therapeutic effects with fewer side effects caused by Tregs. The therapeutic strategies to inhibit Tregs are organized in Table 2.

**Table 2:** Blockade and depletion of Tregs with antibodies and nanoparticles

Type	Load	Delivery Materials	Therapy	Injection	Targeted Protein	Ref
Tregs blockade	Anti-GITR and anti-PD-1	-	in vivo	intratumoral injection	GITR and PD-1	[68]
	Anti-PD-1, anti-PD-L1 and anti-CTLA-4	-	in vitro	-	PD-1 and CTLA-4	[59, 64, 65, 67]
Tregs depletion	Anti-CTLA-4	Iron nanoparticles	in vivo mediated Photothermal therapy	intravenous injection	CTLA-4	[77]
	Anti-CD25 and anti-CCR4	-	in vitro	-	CD25 and CCR4	[72]
	Daclizumab and cyclophosphamide (anti-CD25)	-	in vivo	intravenous injection	CD25	[67, 73]
	Anti-CD36	-	in vitro*, *ex vivo	-	CD36 and PPAR-β	[62, 80]

### Reprogramming T cell exhaustion

T cell exhaustion is one of the T cell dysfunctions that occur during tumor progression and chronic infection [81, 82]. T cell dysfunctions cause disordered tumors, which means the loss in T cell functions [83, 84]. T cell exhaustion occurs after various infections, such as HIV, HCV, and malignancies [85]. The function of IL-2 production is eliminated initially, followed by extinguished TNF-α production, while IFN-ɣ production either endures inactivation or loses its ability by extinguishment [81, 85]. T cells are depleted in the final stage of T cell exhaustion [74]. Exhausted CD8^+^ T cells exhibit a high level of expression of CD43, CD69, and inhibitory receptors; in contrast, they exhibit a low level of expression of CD62L, CD127, and CD122 receptors [81, 85, 86]. However, CD4^+^ T cell exhaustion has not been observed as much as CD8^+^ T cell exhaustion [69].

Some intrinsic factors induce T cell dysfunction. Some transcription factors, such as thymocyte selection-associated HMG BOX (TOX), nuclear factor of activated T cells (NFAT), and member 1 of the nuclear receptor subfamily 4 group A (NR4A), control PD-1 expression and cause T cell dysfunction or exhaustion [87]. TOX is the main regulator of T cell dysfunction progression and CD8^+^ T cell exhaustion during chronic infection. A high expression of TOX can translate constant stimulation and induce CD8^+^ T cell exhaustion. Moreover, NR4A is highly expressed in dysfunctional T cells, and overexpressed NR4A interrupts effector T cell differentiation. Similarly, NFAT is highly expressed in exhausted CD4^+^ and CD8^+^ T cells [88, 89]. The transcriptional progression of CD8^+^ T cell exhaustion is regulated by TOX and NR4A, which are downstream of NFAT [87]. T cell exhaustion is also caused by extrinsic factors. TAMs, cancer-associated fibroblasts, and immunosuppressive cytokines in TME, such as TGF-β or IL-10, induce T cell exhaustion [86, 87]. The upregulation of immune checkpoints and conversion into transcriptional and metabolic molecules are considered T cell dysfunction [90].

#### Avoiding T cell exhaustion

Several therapeutic strategies can be used to avoid T cell exhaustion. One of these strategies is the inhibition of MEK (MEKi) [84, 91]. MEKi enhances the anti-tumor responses of immunotherapy with immune checkpoint inhibitors. Treatment with MEKi increases the gathering of activated CD8^+^ T cells in the TME [84]. Therefore, treatment with MEKi should evolve more effectively. Verma et al. studied the reprogramming of CD8^+^ T cells into memory stem cells with anti-tumor effects using MEKi. MEKi increases anti-tumor responses by preventing exhaustion of CD8^+^ T cells in the TME. MEKi contributes to expanding activated effector T cells, which results in decreased tumor growth in the TME. Furthermore, as MEKi is inhibited, FA metabolism in CD8^+^ T cells increases. Furthermore, enhanced metabolism by inhibiting MEK induces the generation of T_SCM_ in CD8^+^ T cells. T_SCM_ are cells placed between naive and memory T cell populations. T_SCM_ cells produce significantly activated and less exhausted CD8^+^ T cells. Therefore, MEKi treatment generates high anti-tumor immune responses by activating CD8^+^ T cells and preventing T cell exhaustion.

One of the most successful strategies is to use immune checkpoint inhibitors, such as anti-PD-1, anti-PD-L1, and anti-CTLA-4; these alleviate T cell dysfunction or exhaustion and affect chimeric antigen receptor T (CAR-T) cell production positively. CAR-T cells become limited by the influence of TME. However, combining immune checkpoint inhibitors and CAR-T cells increases the therapeutic effects in the TME [83]. Another opinion about using immune checkpoint inhibitors exists. A combination of blocking metabolism has favorable therapeutic effects. Sakuishi et al. observed that only PD-1 expression represents T cell exhaustion imperfectly, but the combination of PD-1 and Tim-3 expression functions as a more accurate marker [89]. Indeed, targeting the Tim-3-Tim-3L and PD-1-PD-L1 pathways can promote a more effective therapeutic strategy in chronic conditions and TMEs [92, 93]. Hung et al. observed that reprogramming the methionine metabolism of tumors can inhibit T cell exhaustion [94]. They analyzed the correlation between T cell exhaustion and methionine, particularly, 5-methylthioadenosine (MTA) and S-adenosylmethionine (SAM). High levels of MTA and SAM were observed to negatively affect T cells. Therefore, methionine metabolite levels are considered potential biomarkers in the TME. Moreover, they observed that SAM and MTA regulate tumorigenesis and CD8^+^ T cell function, *i.e.*, they contribute to the progression of T cell exhaustion and have pivotal roles in tumorigenesis. Hence, they suggested that reprogramming MTA and SAM metabolites could lead to the inhibition of T cell exhaustion and increasing the functions of immune checkpoint inhibitors.

### Reinvigorating T cell exhaustion

**Fig. 7:**
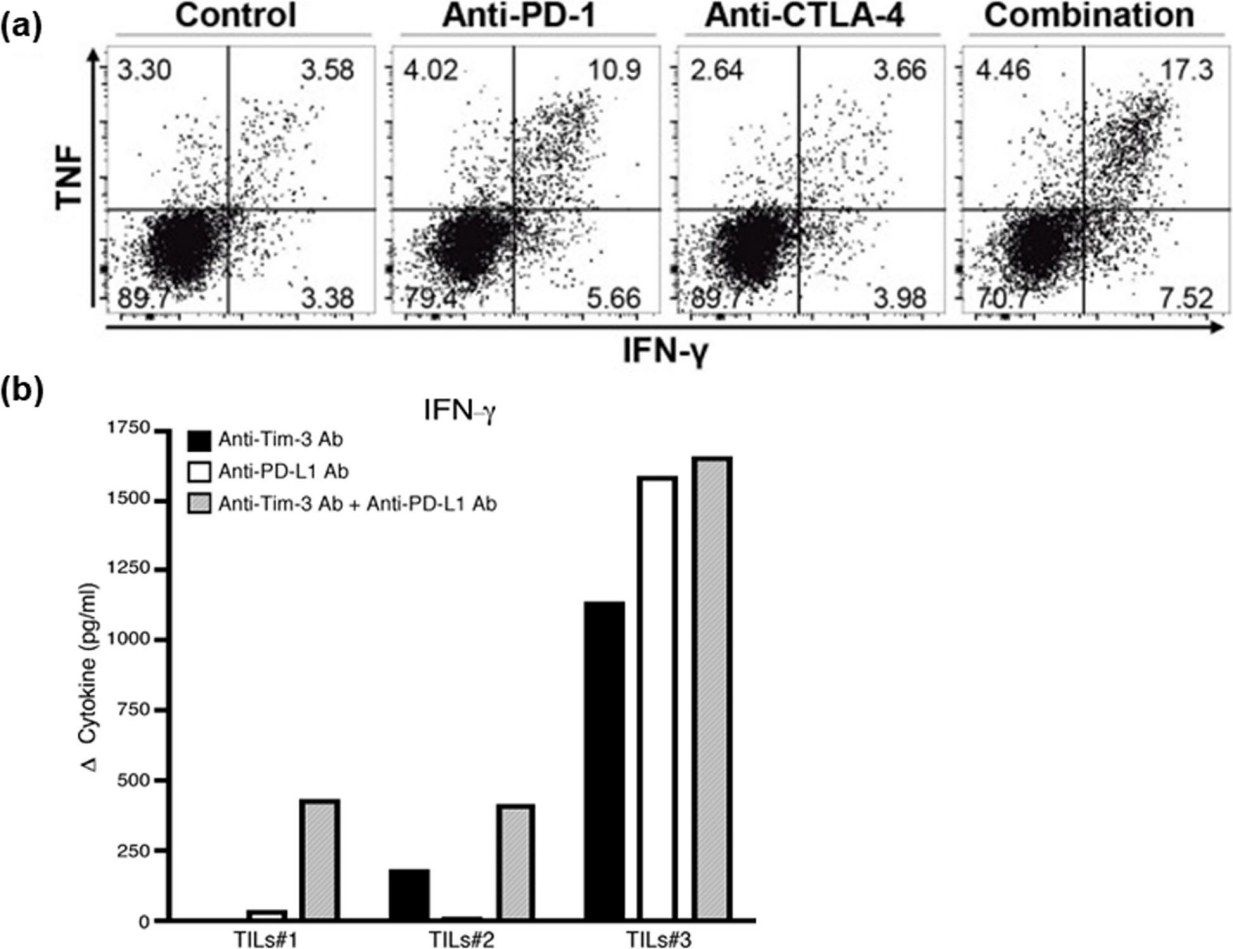
Reprogramming T cell exhaustion with combination therapeutic strategies can increase efficacy. **a** Efficacy of a single PD-1 blockade and combined blockade of PD-1 and CTLA-4 on the production of effector cytokines from CD8.^+^ TILs. (Reproduced with permission from [92] Copyright 2021, Frontiers in Immunology). **b** Blocking the Tim-3 and PD-1 signaling pathways restores IFN-γ production. (Reproduced with permission from [89] Copyright 2021, Frontiers in Immunology)

In addition to inhibiting T cell exhaustion, the reinvigoration of T cell exhaustion is a novel topic for research and has recently been identified as a new therapeutic strategy. Meanwhile, T cell exhaustion interrupts the regulation of inflammations and tumors; modifying the overexpressed pathways can reverse the T cell dysfunctions and reinvigorate immune effects [83]. Kim et al. studied the correlation between PD-1 expression levels and T cell exhaustion [95]. A high level of immune checkpoint inhibitory receptors, such as PD-1 and CTLA-4, implies that T cell exhaustion has progressed even more in the TME [86]. In other words, PD-1 high CD8^+^ T cell accumulation is associated with worse clinical results (Fig. 7a, b) [89, 92]. Therefore, the clinical benefits of PD-1 blockade look forward to enhancing immunotherapy and restoring T cell exhaustion. However, those inhibitory receptors must play a part in the reactivation of exhausted T cells and the system [89].

Recent research has shown that immune checkpoints can interact with metabolic checkpoints. The utilization of glucose limits T cells metabolically, causing reduced mTOR activation of T cells and promoting anti-tumor progression [96]. mTOR is a mammalian rapamycin target, a protein kinase that controls cell growth, proliferation, and survival [88]. Moreover, extracellular glucose availability can be increased by directly blocking PD-L1 in the TME, which results in inhibiting mTOR activation [90].

Currently, drugs used as antibodies to block immune checkpoints, which target PD-1, PD-L1, and CTLA-4, have positive effects on clinical outcomes. For blocking PD-1 and PD-L1, Pembrolizumab, Nivolumab, and Pidilizumab were used in the first phase of clinical trials for the therapeutic interventions of cancer patients [97]. Although the molecular mechanisms of PD-1 regulate T cell exhaustion. Various mechanisms are combined with PD-1 by using other inhibitory receptors or monoclonal antibodies [83]. NR4A has been revealed as a major mediator for T cell function, and lack of NR4A results in the downregulation of PD-1 expression. Therefore, inhibiting NR4A functions and immune checkpoints is essential for tumor immunotherapy, which could reinvigorate T cell functions in the TME [89, 98, 99]. The therapeutic strategies for reprogramming T cell exhaustion are organized in Table 3.

**Table 3:** Reprogramming pathways or metabolism to avoid and reinvigorate CD8^+^ T cell exhaustion

Type	Load	Delivery Materials	Therapy	Injection	Targeted Protein	Ref
Avoiding T cell exhaustion	MEKi	**-**	ex vivo	-	MEK	[84]
	MEKi with selumetinib and anti-CTLA-4	HPMC	in vivo	subcutaneous injection, oral administration, and intraperitoneal injection	-	[91]
	Anti-PD-1 and anti-Tim-3	-	in vitro	-	PD-1, PD-L1, Tim-3, and Tim-3L	[89, 92, 93, 99]
	MTA and SAM	-	in vitro*, *ex vivo	-	CD2, CD3, and CD28	[94]
Reinvigorating T cell exhaustion	Anti-PD-1 and anti-CTLA-4	-	ex vivo	-	CD28, CD3, PD-1, and CTLA-4	[86, 89, 92, 95]
	Pembrolizumab, Pidilizumab and Nivolumab	-	ex vivo*, *in vitro	-	PD-1 and PD-L1	[97]
	NR4A and NFAT inhibitor	-	in vivo	intravenous injection	NR4A and NFAT transcription factor	[98]

### Increasing T cell infiltration

The tumor infiltrating T cells play essential roles in tumor immunity. The infiltration of T cells is regulated by immune checkpoints, such as CTLA-4, PD-1, and PD-L1 [93]. Significant infiltration of T cells converts “cold tumor” into “hot tumor”, which is related to the effects of immune checkpoint inhibitors and is considered a biomarker to determine the degree of T cell infiltration or activation [100–102]. Immune cells affect the prolonged survival of patients and improve the immune responses by tumor cells [100]. In contrast, the lack of immune cells causes interruption in immunotherapy. Furthermore, another therapeutic method exists, which uses the increased infiltration of T cells called CAR-T cells.

### Immune checkpoint inhibitors

Recent studies observed that the monotherapy with anti-PD-1 or anti-CTLA-4 antibodies can induce anti-tumor effects in prolonged survival of cancer patients [101, 103–105]. However, most cancer patients did not respond to this monotherapy. Therefore, improved therapeutic strategies were investigated [106]. A recent study was based on CD8^+^ T cells administering anti-PD-1 monotherapy or in combination with anti-CTLA-4. It improves and restores the efficacy of T cell activity and infiltration in the TME [101–107].

As mentioned earlier, MEKi increases the anti-tumor effects of T cells. However, most studies investigated the effects of only MEKi. Poon et al. researched the combination of MEKi and anti-CTLA-4 antibodies for tumor therapy [91]. They primarily attempted to prime the intracellular T cells using pre-treatment with selumetinib. Treatment with selumetinib increased T cell proliferation; however, it did not significantly impact the anti-tumor activity. Thus, they considered the combination of MEKi, which was selumetinib and anti-CTLA-4. It enhanced anti-tumor activity in the TME and synergized the immunotherapy better than using each alone.

Another therapeutic strategy, a combination of PD-L1 and TGF-β blockades, was investigated by Mariathasan et al*.* [108]. The lack of immune responses is associated with TGF-β signaling in fibroblasts, which occurs in cancer patients who do not have sufficient number of CD8^+^ T cells. TGF-β is the central mediator to promote angiogenesis and metastasis in the late stage of tumor progression in the TME. Mariathasan et al. observed that the therapeutic combination of TGF-β blockade and anti-PD-L1 pathways can decrease TGF-β signaling in the TME and reshape the TME by enhancing anti-tumor activity by disrupting T cell infiltration. As a result, this treatment significantly increased the number of effector CD8^+^ T cells, while Tregs remained unaffected. Thus, the restriction of TGF-β signaling can enhance anti-tumor immune responses of anti-PD-L1 and cause tumor regression.

### Chimeric antigen receptor T cells

CAR technology is creative immunotherapy that involves genetic modification of T cells [109]. It uses the innate ability of the immune system to selectively encounter tumor cells in an MHC-independent manner [110]. CAR is designed to reprogram lymphocytes, particularly T cells, to recognize and remove cells that express specific antigens [109]. CAR consists of the extracellular antigen binding domain, hinge region, transmembrane domain, and intracellular signaling domains [109–111]. CAR can identify specific antigens with the antigen binding domain, which exists in the extracellular space with the antigen recognition region [110, 111]. The hinge region, also called the spacer region, is the extracellular space that extends the binding units from the transmembrane domain [111]. Moreover, it enables the antigen binding domain to administer the target. The transmembrane domain functions as a connection between CAR and T cells [110]. Endodomain activates T cells after CAR binding with targeted antigens. T cell infiltration using the T-cell receptor (TCR) has some limitations: 1) the low affinity of TCR for targeted cancer and 2) the limitation of the activation and cytotoxicity of T cells, which target tumor cells [112].

However, CAR can complement these limitations of TCR. CAR leads the precise response and contains a combination of signaling or costimulatory molecules [113]. There are various challenges to and several successful results of immunotherapy with CAR cells [107, 112–117]. Recently, therapeutic strategies have focused on modifying CAR-T cells to produce immune checkpoint inhibitor antibodies, which incorporate switch receptors for CAR-T cells that target inhibitory receptors, and interrupt inhibitory receptors on T cells by CRISPR-Cas9 [114]. The most successful study targeted CD19 with CAR. CD19 was the initial target because it is frequently expressed at high levels in B cell malignancies [112]. As a result, the CD19-CAR-T cell has been used to treat patients successfully, with a response ratio of 60% [116]; however, side effects of the CD19-CAR-T cell also exist, which include B cell aplasia.

Therefore, a new approach has been studied recently, which is a combination of CAR-T cell and immune checkpoints, such as PD-1 antagonists [112]. Chong et al. researched the combination of CAR-T cells and anti-PD-1. It resulted in anti-tumor responses, such as the expansion of CAR-T cells to respond effectively against cancers. Their experiments indicated that the PD-1-PD-L1 pathway is critical, and blockade of the pathway increases immune responses in CAR-T cell immunotherapy [115]. Future research on CAR-T cells should focus on overcoming the low-oxygen environment of the TME, improving the functions of CAR-T cells against tumor cells, and activating the innate anti-tumor responses by CAR-T cells in the TME [114, 117]. Indeed, the combined therapeutic strategies, such as using an immune checkpoint blockade, can enhance the clinical responses in the TME [107, 112–117].

### Targeting metabolic pathways

T cells influence activation and differentiation by undergoing metabolic changes [118]. Tumor cells also proceed with the growth, proliferation, or metastasis based on the cell metabolism in a low-oxygen TME [118, 119]. Moreover, tumor cells regulate the differentiation of immune cells in the TME via the metabolites of tumor cells, which can encourage tumor growth and Tregs and inhibit effector T cell infiltration and immune responses [118, 120]. Therefore, various types of research have recently focused on the reprogramming of T cell metabolism in the TME. The three T cells primary metabolism in the TME are glucose, lipid, and amino acid metabolism. The reprogramming of these three pathways needs to be studied.

#### Glucose metabolism

T cells can generate adenosine triphosphate (ATP) through glycolysis and oxidative phosphorylation, and ATP promotes T cell activation [121]. However, tumor cells accelerate glycolysis under aerobic conditions and support their rapid growth and differentiation [122, 123]. This phenomenon is called the “Warburg effect” [124]. The Warburg effect is the utilization of fermentation in aerobic conditions and is characterized by increased glucose intake and consumption, reduced oxidative phosphorylation, and lactic acid production [125].

Glucose is metabolized in the following three pathways: tricarboxylic acid (TCA) cycle, glycolysis, and pentose phosphate pathway [80, 121]. Pyruvate dehydrogenate kinase 1 (PDK1) is a major regulator in glucose metabolism [126]. PDK1 stimulates lipopolysaccharide (LPS), activates macrophage inflammatory responses, and induces pro-inflammatory cytokines from M1 in the TME. Pro-inflammatory cytokines induce glycolytic genes, such as phosphofructokinase 1, hexokinase 2 (HK2), and pyruvate kinase M2 (PKM2) [126, 127]. HK2 is a glucose receptor that can knock down PDK1 through phosphorylation [126]. In the stage in which fructose-6-phosphate changes into fructose-1,6-bisphosphate, 6-phosphofructo-2-kinase/fructose-2,6-bisphosphatase isoenzymes stimulate IFN-γ and LPS, which induce the production of fructose-2,6-biophosphate [127]. Thus, glycolysis in M1 increases. Furthermore, during the conversion of phosphoenolpyruvate into pyruvate, PKM2 induces hypoxia-inducible factor (HIF-1α) [80]. HIF-1α promotes tumor growth by expressing vascular endothelial growth factors [127]. Therefore, targeting enzymes in glycolysis is considered a potential therapeutic strategy. Glucose metabolism is associated with TAMs [80]. Polarized macrophages (M1 and M2) depend on metabolism in the TME. In the TME, TAMs function as M2-like macrophages *i.e.,* shows immunosuppressive activity. TAMs require more glucose for increased energy to continue mitochondrial respiration [128]. M1 relies on a decreased level of glycolytic metabolism; in contrast, M2 relies on increased glycolytic flow [121]. Thus, in cancer therapy, inhibitory glycolysis and TAMs-targeted therapeutic strategies are the main focus.

Primarily, the use of resveratrol has recently been investigated. The focus has been on its anti-tumor activities and effects on glucose metabolism. Jung et al*.* revealed that resveratrol induces the actual inhibition of glucose metabolism in the TME. The increased number of reactive oxygen species (ROS) is a common feature of tumor cells. As a result, tumor cells decrease with dose-dependent administration of resveratrol. Glucose uptake by tumor cells is suppressed with regulated glucose metabolism, ROS levels are reduced, and glucose uptake is also suppressed [129]. One of the metabolism reprogramming pathways is the use of anti-PD-L1 therapy [128]. Jia et al*.* studied the balance of glucose metabolism, which is the glycolysis and oxidative phosphorylation of tumor cells, and developed the dual-responsive polyplexes for robust co-delivery of resveratrol and anti-PD-L1. The co-delivery polyplexes were mPEG-PLA-PHis-ss-PEI polyplexes, which could enhance PD-L1 silencing responses by balancing the glucose metabolism of tumor cells. Resveratrol and anti-PD-L1 induce the reductive environment in TME by interrupting glycolysis and activating oxidative phosphorylation to build the balancing glucose metabolism. Thus, the balancing pathways produce less immunosuppressive cells in the TME.

#### Lipid metabolism

The reprogramming of the lipid metabolism is vital in forming the TME. Increasing lipid metabolites results in immunosuppression and tumorigenesis in the TME [130]. Lipid metabolisms include FA metabolism, cholesterol metabolism, arachidonic acid metabolism with Prostaglandin E2, and transduction of PPAR signal [131]. Acetyl-CoA is synthesized in the TCA cycle from FAs and cholesterol metabolism [132]. An essential characteristic is metabolism alteration. Lipid metabolism alteration influences the anti-tumor activities of immune cells and cause immune elusion. The reprogramming of lipid metabolism can satisfy the requirement for energy and nutrient supply to involve a sharp growth of the tumor [131, 132]. The uptake and synthesis of lipids in the TME become different from normal cells owing to the reprogramming of lipid metabolism [132]. The reprogramming of lipid metabolism can affect tumor and immune cells or T cells [131].

One of the most effective lipid metabolisms reprogramming pathways is the blocking of lipid uptake. Tumor cells compete with other cells to obtain the oxygen and nutrients in the TME. Moreover, the FA pathway aids in maintaining malignant potential and become targets of tumor cells [125]. FA is necessary to proliferate tumor cells; inhibiting availability can result in therapeutic strategies. Several methods to restrict the availability of FAs are increasing FA degradation through oxidation, blocking FA synthesis, or reducing FA uptake. CD36 is the receptor on the tumor cells that promotes lipid uptake from extracellular conditions and functions as a marker of metastasis [133]. High expression of CD36 is relevant to high free FA, which induces metastasis and activation of the TGF-β signaling pathway [131]. Therefore, the targeting CD36 can inhibit the absorption of tumor cells and metastasis [134]. FA synthesis is upregulated by the expression levels of enzymes, such as the ATP citrate lyase (ACLY), Acetyl-CoA carboxylase, and FA synthase [125, 135].

Cholesterol is a necessary lipid in cell membranes and the foundation of cancer cell proliferation. Cholesterol homeostasis is essential in maintaining cell membrane functions [132]. FA and cholesterol metabolism is regulated by sterol regulatory element-binding proteins (SREBP) [135]. The activation of tumor growth signaling pathways (PI3K/AKT and RAS/MARK pathways) stimulates glucose uptake and usage in lipid synthesis through SREBP activation [131]. Moreover, tumor cells with RAS mutation enhance cholesterol synthesis by increasing phosphoenolpyruvate carboxykinase 1 (PCK1) [132]. ACLY, which produces Acetyl-CoA and is regulated by SREBP1, is activated by fructose-6-phosphate [134]. Furthermore, cholesterol is synthesized from acetyl-CoA by 3-hydroxy-3-methylglutaryl coenzyme A reductase (HMGCR), whose levels function as the main component of lipid production [131, 134]. One of the ways to reprogram cholesterol metabolism is targeting cholesterol biosynthesis. Cholesterol biosynthesis can be targeted using an inhibitor of HMGCR. Statins, the HMGCR inhibitors, induce the feedback responses to decrease cellular cholesterol levels and apoptosis to maintain homeostasis [131, 136].

### Amino acid metabolism

Amino acids (AAs) are divided into two types: essential amino acids (EAAs) and non-essential amino acids (NEAAs) [137]. Tumor cells are addicted to specific AA and are upregulated abnormally. AAs, which are under genotoxic, oxidative, and nutritional stress, prolong the survival and proliferation rate of tumor cells [138]. AA uptake and metabolites may become essential contributors of tumor growth in the TME [130]. AA metabolism primarily includes serine, glycine, glutamine, and branched-chain amino acid metabolism [130]. Serine and glycine are connected biosynthetically. Serine is a one-carbon source in nucleotide synthesis and DNA methylation [138]. It has a pivotal role in the rapid proliferation and growth of tumor cells. Therefore, increased levels of serine mean faster proliferation and growth of cancer and vice versa [130, 138]. Serine and glycine provide the precursors necessary to synthesize vital proteins, nucleic acids, and lipids necessary for tumor cell growth and homeostasis [138]. Therefore, the restriction of serine and glycine intake can decrease tumor growth and prolong survival time [130].

Glutamine is a NEAA; however, tumor cells depend on it to survive through the MAPK/ERK pathway in the TME [127, 139]. In other words, glutamine is a selective EAA for tumor cells. Thus, tumor cells increase glutamine uptake and utilization in the TME [140]. It synthesizes AAs, lipids, and nucleic acids as significant nitrogen and carbon sources. Furthermore, glutathione can be synthesized through carbon and nitrogen donation. Blocking single glutamine transporters during importing into tumor cells or inhibiting glutaminase can prevent the growth and proliferation of tumor cells [139, 140]. Arginine is an EAA and a necessary material for protein biosynthesis [141, 142]. Glutamine results in the expression of leucine and arginine [137, 142]. Therefore, the carbon and nitrogen, which are used to synthesize proteins, are provided by glutamine [127, 140]. Arginine stimulates mTOR by activating mTORC1, an essential regulator of G1 cell cycle progression for cell division and replication [127, 141]. The activation of mTORC1 induces the growth and expansion of tumor cells in the TME [139]. Thus, suppressing and inhibiting mTOR signaling can decrease the survival rate and growth of tumor cells [143]. Large-neutral amino acid transporter 1 is the representative suppressor of mTOR signaling. Indeed, the restriction of cationic amino acid transporter 1, which is an arginine transporter, can decrease arginine uptake and prevent its further signaling.

## Conclusions

Tumor cells stimulate major molecular, cellular, and physical alterations in their host tissues. The TME is a unique environment that is developed by the tumor and controlled by tumor-induced interactions with host cells during tumor progression. Monotherapy exhibits low tumor treatment responses; thus, immunotherapy and biomaterials have been combined. One of the combination immunotherapies is the reprogramming of the TME to increase the tumor treatment responses. In this review, we introduced the methods of reprogramming TME: macrophage polarization, inhibiting Tregs, reprogramming T cell exhaustion, T cell infiltration, and targeting metabolic pathways. Macrophage polarization, which converts M2 into M1, is controlled by TLR agonists with cytokines, such as anti-PD-1 and anti-CD47 with anti-SIRPα to block CD47-SIRPα. To inhibit Tregs, which promote tumor growth by suppressing the immune response, a combination of anti-GITR and anti-PD-1 was effectively used to block Tregs and target CTLA-4 and clusters of differentiation to deplete Tregs. Reprogramming T cell exhaustion was conducted in two ways: avoiding T cell exhaustion and reinvigorating exhausted T cells. A few successful strategies for reprogramming T cell exhaustion and infiltrating T cells include MEKi, CAR-T cells, and immune checkpoint inhibitors. Moreover, targeting metabolic pathways is being researched currently. Three main types of metabolism existed: glucose, lipids, and amino acids. Targeting the TCA cycle is the most effective therapeutic method to increase tumor treatment responses in the three types of metabolism. In conclusion, the combination of therapeutic strategies resulted in more effective responses than monotherapy. Indeed, biomaterials, such as nanoparticles or hydrogels, aid in increasing the efficacy of drugs or immunotherapies. With the use of these combination therapies, nanoparticles, and hydrogels, more effective therapeutic strategies and applications with several drugs would increase future anti-tumor immune responses from patients.

## Data Availability

Not applicable.

## Abbreviations

PEG: Polyethylene Glycol
TME: Tumor microenvironment
NK cells: Natural Killer cells
DCs: Dendritic cells
Tregs: Regulatory T cells
M1: Macrophage phenotype 1
M2: Macrophage phenotype 2
CD: Cluster of Differentiation
Th1 CD4^+^ cells: The helper CD4^+^ T cells
IL: Interleukin
IFN: Interferon
TNF: Tumor Necrosis Factor
TAMs: Tumor Associated Macrophages
PD-1: Programmed death-1 receptor
MHC: Major Histocompatibility
APCs: Antigen presenting cells
TLR: Toll-like receptor
LNPs: Lignin nanoparticles
R848@LNPs: LNPs loading R848
CD: β-Cyclodextrin
CDNP-R848: R848 loaded on CD nanoparticles
PPAR: Peroxisome proliferator-activated receptor
mTORC1: mTOR complex 1
IONPs: Iron-oxide nanoparticles
PTT: Photothermal therapy
TfR: Transferrin receptor
PIC: Poly (I:C)
FP-NPs: Nanoparticles composed of amino-modified Ferumoxytol-NH_2_ surface functionalized with Poly (I:C)
CNs: Chitosan nanoparticles
SIRPα: Signal regulatory protein alpha
CALR: Calreticulin
nMOF: Nanoscale metal–organic framework
FAO: Fatty acid oxidation
TOX: Thymocyte selection-associated HMG BOX
NR4A: Nuclear receptor subfamily 4 group A
NFAT: Nuclear factor of activated T cells
MEKi: Inhibition of MEK
CAR: Chimeric antigen receptor
MTA: Methylthioadenosine
SAM: Adenosylmethionine
mTOR: Mammalian target of rapamycin
TCR: T-cell receptor
TCA: Tricarboxylic acid
PDK1: Pyruvate dehydrogenate kinase 1
HK2: Hexokinase 2
PKM2: Pyruvate kinase M2
HIF-1α: Hypoxia-inducible factor
ROS: Reactive oxygen species
FA: Fatty acid
ATP: Adenosine triphosphate
ACLY: ATP citrate lyase
SREBP: Sterol regulatory element-binding protein
PCK1: Phosphoenolpyruvate Carboxykinase 1
HMGCR: 3-hydroxy-3-methylglutarylcoenzyme A reductase
AA: Amino acid
EAA: Essential amino acid
NEAA: Non-essential amino acid
